# Complete remission of depression and anxiety using a ketogenic diet: case series

**DOI:** 10.3389/fnut.2024.1396685

**Published:** 2024-05-14

**Authors:** Lori Calabrese, Rachel Frase, Mariam Ghaloo

**Affiliations:** ^1^Innovative Psychiatry, LLC, South Windsor, CT, United States; ^2^Trinity College, Hartford, CT, United States

**Keywords:** ketogenic metabolic therapy, KMT, ketogenic diet (KD), metabolic dysfunction, depression, anxiety, case report

## Abstract

**Background:**

There is little data that describe the use of ketogenic metabolic therapy to achieve full remission of major depression and generalized anxiety disorder in clinical practice. We present a retrospective case series of three adults with major depression and generalized anxiety disorder with complex comorbidity, treated with personalized ketogenic metabolic therapy, who achieved complete remission of major depression and generalized anxiety disorder and improvements in flourishing, self-compassion, and metabolic health.

**Methods:**

Three adults, ages 32–36, with major depression, generalized anxiety, other anxiety disorders, and comorbid psychiatric conditions were treated for 12–16 weeks with personalized whole food animal-based ketogenic metabolic therapy (1.5:1 ratio) in a specialized metabolic psychiatry practice. Interventions included twice-weekly visits with an experienced ketogenic registered dietitian; daily photo journaling and capillary blood BHB/glucose/GKI monitoring; virtual groups; family/friends support; nature walks and talks several times per week, and community building. Successful adoption of the ketogenic diet was defined as the achievement and maintenance of capillary BHB ≥ 0.8 mmol/L and GKI < 6. Remission was assessed by GAD-7 and PHQ-9, and quality of life was assessed subjectively and with validated scales for flourishing and self-compassion. Metabolic health was assessed by laboratories/biometric measures.

**Results:**

Two patients achieved remission of major depression (PHQ-9 ≤ 4) and generalized anxiety (GAD-7 ≤ 4) within 7 weeks of therapeutic nutritional ketosis; one required 12 weeks. Anxiety responded and remitted more quickly than major depression. Flourishing and self-compassion increased steadily. Patients lost 10.9 to 14.8% of their initial body weight within 12 weeks and improved metabolically; one achieved optimal metabolic health.

**Conclusion:**

Complete remission of major depression and generalized anxiety disorder occurred within 7–12 weeks of therapeutic nutritional ketosis during treatment with a personalized animal-based ketogenic diet (ratio 1.5:1) in adults with complex comorbid depression and anxiety engaged in a specialized metabolic psychiatry program.

## Introduction

Emerging brain-based research in psychiatry and neurology has focused on identifying fundamental metabolic disturbances within neurons and throughout the body involving insulin resistance, inflammation, oxidative stress, and alterations of the gut microbiome (1). All four of these fundamental metabolic disturbances are present in major depression (2), and underlying anxiety disorders (3) and can be directly modulated through the use of ketogenic metabolic therapy (KMT) (4).

As psychiatric disorders have risen over the past several decades, the prevalence of metabolic syndrome has sharply increased, with only 12.2% of U.S. adults meeting the criteria for optimal metabolic health, leaving 87.8% metabolically compromised (5, 6).

Metabolic syndrome affects almost a third of individuals with major depression (7). It is a significant contributor to their morbidity and mortality (8) and is rooted in impaired glucose metabolism and utilization. Insulin resistance has been well described in many tissues, including the brain (9), where it is being investigated as a link between metabolic health and mental health conditions. Preclinical models demonstrate that glucose intolerance is directly associated with anxiety and that insulin resistance triggers depressive behaviors (9). In brain tissue, insulin resistance results in cerebral glucose hypometabolism and a vicious cycle of unmet energy needs (10). In human studies, cerebral glucose hypometabolism is a feature of major depression (11, 12) and generalized anxiety disorder (GAD) (13).

KMT, also known as the therapeutic ketogenic diet, or KD, is a low carbohydrate, moderate-protein, high-fat diet that supports a fundamental metabolic shift from glucose to ketone bodies as the primary fuel source (14). Classic KMTs are formulated with strict macronutrient ratios, most commonly 4:1 and 3:1 (fat: protein + carbohydrates), and have demonstrated efficacy in intractable epilepsy and genetic disorders. More recently, modified classic KMTs with lower macronutrient ratios of 2.5:1, 2:1, and 1.5:1 have been utilized in research and clinical practice (15, 16). These allow more variety in the diet, meet micronutrient needs except vitamin D (17), and are easier to sustain for extended periods of time.

KMT exploits the body’s natural ability to produce ketone bodies (d-beta-hydroxybutyrate (BHB), acetoacetate, and acetone) in the liver from fatty acids by keeping carbohydrate consumption very low. Acute and sustained production of ketone bodies produces a fundamental shift in fuel energetics within cells, particularly neurons, which can radically re-route and quickly rely on readily available BHB and acetoacetate for cellular energy (18). Ketone bodies also increase vascular density at the blood–brain barrier, which can strikingly increase the availability of ketone bodies for brain energy metabolism by 40-fold (19). Ketones are a preferred energy source in the CNS (20) and neurons will choose ketones over glucose when available.

Nutritional ketosis (10) using a KMT is a natural, not pathological, state (21) where the body’s energy and protein synthesis needs are met with a high-fat/moderate-protein/low-carbohydrate diet, resulting in sustained elevations of serum ketones and fatty acids and normal glucose without acidemia. In both acute and long-term nutritional ketosis, ketone bodies have a number of biological effects that directly change the brain’s cellular energy status (15), increase mitochondrial density (22), and improve mitochondrial morphology, which has been shown to be altered in mood disorders (23, 24). Mitochondrial abnormalities have also been postulated to be responsible for changes in synaptic function and neuroplasticity, potentially associated with symptoms of depression and anxiety (19).

Recent research shows that a ketogenic diet (KD) reduces neuronal firing rates, modulates ion channels and cell signaling cascades, and stimulates the biochemical synthesis and neurotransmission of GABA by inhibiting glutamate decarboxylase, a major inhibitory neurotransmitter involved in neuronal firing and anxiogenesis (25, 26). BHB activates the transcription of antioxidant-related genes by inhibiting histone deacetylases, triggering long-term adaptive changes in gene expression. In addition, at physiologic concentrations, ketone bodies reduce neuroinflammation through direct action at G-protein coupled receptors (25). KD also favorably alters the gut microbiome (27). Perhaps most importantly, KD directly increases NAD+, which reduces reactive oxygen species and increases mitochondrial ATP production. It is also utilized as a substrate for sirtuins and PARP enzymes associated with DNA repair and longevity. A sustained increase in NAD+ may underlie the pleiomorphic benefits of KMT across multiple neuropsychiatric conditions (28). In terms of the frequent abnormal alarms set off in the amygdala during anxiety, nutritional ketosis may provide an acute and long-term intervention to reduce generalized anxiety, panic attacks, obsessive doubt, and symptoms of post-traumatic stress disorder (PTSD). For apathy, anhedonia, amotivation, and abulia seen in major depression, therapeutic nutritional ketosis may provide higher and more sustained intraneuronal energy and repair (29, 30).

There is no published data that describes the implementation and use of personalized KMT for adults in real-world clinical practice who present with major depression comorbid with GAD and complex psychiatric comorbidity.

The aim of this case series is to examine the response to the treatment of major depression and generalized anxiety with whole-food animal-based personalized KMT in adults with complex psychiatric comorbidity and varied metabolic status. We conducted a retrospective review of three cases from our Metabolic Psychiatry Registry that demonstrate a consistent response and remission of major depression and generalized anxiety among patients who are psychiatrically and metabolically complex, despite differences in the initiation and adoption of KMT, and varied metabolic dysfunction. We describe the evaluation process and prescription of KMT, baseline metabolic workup and monitoring, elements that fostered treatment engagement and adherence, and challenges encountered during 12 weeks of KMT. We correlated capillary BHB/GKI with time to the remission of major depression and GAD and the achievement of metabolic health.

## Case presentations

We present individual case descriptions with time to response and remission, clinical challenges during KMT, and metabolic outcomes. Response was assessed quantitatively using PHQ-9, GAD-7, and correlated with BHB drawn from capillary blood and glucose–ketone index (GKI) using Keto-Mojo® GK+ Blood Glucose and β-Ketone Dual Monitoring System (ketone/glucose correlation coefficients to serum of 0.9927/0.9974) to determine the length of time to response and remission. Improvement in quality of life was assessed by qualitative reports during clinical visits and quantitatively using Self-Compassion Scale (SCS) and Flourishing Scale. Additional rating scales were used throughout treatment to assess symptomatic response during KMT to comorbid psychiatric conditions.

It is important to stress that in our therapeutic intervention (see Supplementary Data). KMT was used as a medical prescription, with a properly formulated, individualized ketogenic diet offered in conjunction with multiple clinical supports and lifestyle therapies, including sleep, circadian rhythms, movement, community building, friends, and family supports provided by the program, small group nature walks and talks with other patients, and the registered dietitian and psychiatrist, psychiatric follow-up, and metabolic monitoring. Clinician contact was frequent, in person, virtually, through digital tools, and outdoors in nature.

Metabolic health was assessed by interval measures of relevant laboratories, % body fat, visceral fat level, and blood pressure.

### Case 1

Case 1 is a 32-year-old unemployed married man. He had a lifelong history of previously unrecognized and untreated recurrent major depression, as well as GAD, obsessive-compulsive disorder, trypanophobia, and binge eating disorder. He experienced prominent inattention and distractibility since childhood; an adequate prior trial of atomoxetine 100 mg po qd for 1.5 years was somewhat effective; and lisdexamfetamine 70 mg was somewhat effective. He had long declined consideration of SRIs, SNRIs, other antidepressants, and buspirone. He was unaware of the degree to which his complex symptoms had pervasively affected his functioning and quality of life, resulting in his inability to sustain employment, financial insecurity, and adverse interpersonal relationships. Medical history was notable for hypothyroidism with negative antibodies treated with levothyroxine 25 mcg, declined treatment for known hypertension, and frequent snoring without evaluation for obstructive sleep apnea. Metabolically, he was obese (BMI 34.7 kg/m^2^), with a percent body fat of 36.1%, with a history of muscle cramping during exercise and longstanding untreated essential hypertension (BP 138/102); labs revealed a dyslipidemic profile with TG 241 mg/dL; TG/HDL ratio was 6; and AST/ALT were elevated at 44/82 mg/dL. Fasting glucose was 82 mg/dL.

Family history was notable for anxiety, thyroid dysfunction, and hypertension in first-degree relatives.

**Figure 1:**
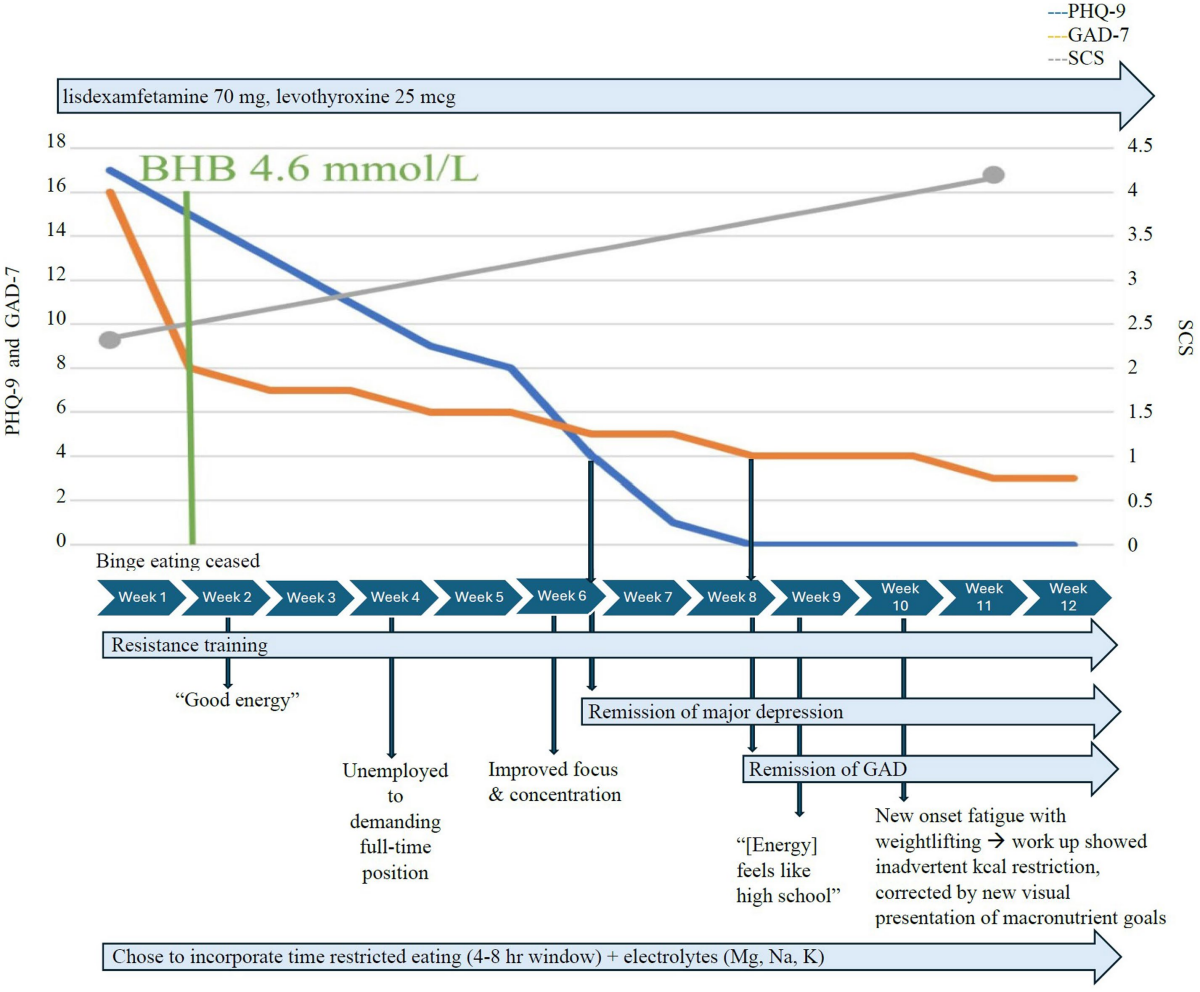
Case 1 KMT timeline.

At the initiation of KMT 1.5:1, Case 1 chose to incorporate time-restricted eating and consumed two meals per day within a 4–8 h eating window. He achieved therapeutic nutritional ketosis with a mean serum BHB of ≥0.8 mmol/L, GKI < 6 within a couple of days, and high average serum BHB levels of 4.6 mmol/L within 1 week (Figure 1), without adverse effects. He maintained adherence without the muscle cramping he had experienced with exercise before KMT, due to close attention to electrolyte needs and supplementation during KMT. Vitamin D deficiency (26 ng/mL 25-OHD3) was treated with vitamin D3/K2 5,000 IU qd. Generalized anxiety response (GAD-7 decreased from 16 to 8) within 1 week and completely remitted 6 weeks later. The initial PHQ-9 of 17 indicated moderately severe depression. Depressive symptoms completely remitted (PHQ-9 ≤ 4) within 5 weeks of consistent therapeutic nutritional ketosis. Binge eating ceased within days of KMT initiation, and he reported that he “no longer gets over hungry,” and that he “no longer eat[s] without realizing [he’s] eating.” The SCS increased from 3 to 4.6 over 4 weeks. The Flourishing Scale increased significantly from 44 to 53 at 14 weeks; the 12-week data were missing. He reported increased mental focus, increased energy, renewed confidence, and motivation to return to work. Within 4 weeks of initiating KMT, he secured a demanding full-time position exceeding his previous experience; after 8 weeks, he was given additional responsibilities, handled them well, and began three online college courses.

At week 10, he reported fatigue during weightlifting but declined to obtain labs. Photo journaling revealed only one meal a day frequently, which was inadequate to meet his protein needs and macronutrient/micronutrient goals. The inquiry revealed he intentionally restricted his intake due to fear of slowing down his body fat loss. KMT macronutrient needs were reviewed for his current lean body mass and overall body composition, and presented in a new visually appealing way. He was reassured and he quickly increased his intake to a minimum of two meals per day with increased fats as recommended. By 12 weeks, obsessive thoughts lysed (YBOCS 1), and “anxiety was almost gone.” Interpersonal relationships were improved.

Metabolically, Case 1 lost 36.9 lbs./16.8 kg over 12 weeks, BMI decreased from 34.7 to 29.6 kg/m^2^, % body fat decreased from 36.1 to 28.7%, without loss of lean body mass, and blood pressure normalized from 136/102 to 116/81.

### Case 2

Case 2 was a 36-year-old married man with a lifelong history of mood dysregulation, irritability, and trauma from adverse childhood experiences and recent work experiences. He had a history of childhood-onset generalized anxiety, panic disorder, and PTSD, as well as recurrent major depression, which was moderately severe and persistent. His anxiety was unrecognized and untreated. Although he did not have a history of mania, hypomania, or mixed states, he had been treated throughout childhood and adolescence with sertraline 50 mg (ineffective), duloxetine 60 mg (agitation), escitalopram 20 mg (diaphoresis), lamotrigine 300 mg (poor school performance), divalproex ER 1,500 mg (tremor, sluggishness, and weight gain), oxcarbazepine 1,200 mg (fatigue), olanzapine (hunger and weight gain), methylphenidate (ineffective), amphetamine-dextroamphetamine mixed salts 20 mg, and lisdexamfetamine (tachycardia). He discontinued all psychiatric treatment at age 19, but remained symptomatic for 15 years. Recent work-related trauma was unresponsive to psychotherapy and prompted him to present for evaluation and treatment; in addition to major depression and three concurrent anxiety disorders, he met DSM-V criteria for ADHD, a combined type, which had not been previously recognized. Medical history was notable for juvenile ankylosing spondylitis, hyperlipidemia, obstructive sleep apnea, cholecystitis, vitamin B12 deficiency, and vitamin D deficiency. There were no medications other than supplemental vitamin D3 (2,000 IU po qd). Metabolically, he was overweight with a BMI of 28.7 kg/m^2^, % body fat 26.1, elevated visceral fat level 10, HS-CRP 2.5, total cholesterol 247 mg/dL, and LDL 174 mg/dL. Fasting insulin, HOMA-IR, and blood pressure were WNL.

Family history included bipolar disorder, depression, anxiety, ADHD, hyperlipidemia, hypertension, colon cancer, and breast cancer.

He adopted KMT within less than a week, increased vitamin D3/K2 to 5,000 IU po qd, added magnesium glycinate 250–350 mg po qd, and replaced his sugar-containing electrolyte drink with one free of added sugars. However, he initially struggled to meet his daily goals for fat consistently, exercised heavily, and reported new-onset fatigue during exercise. Serum ketones were variable and fluctuated between 0.2 and 1.8 mmol/L throughout the first 8 weeks of KMT.

**Figure 2:**
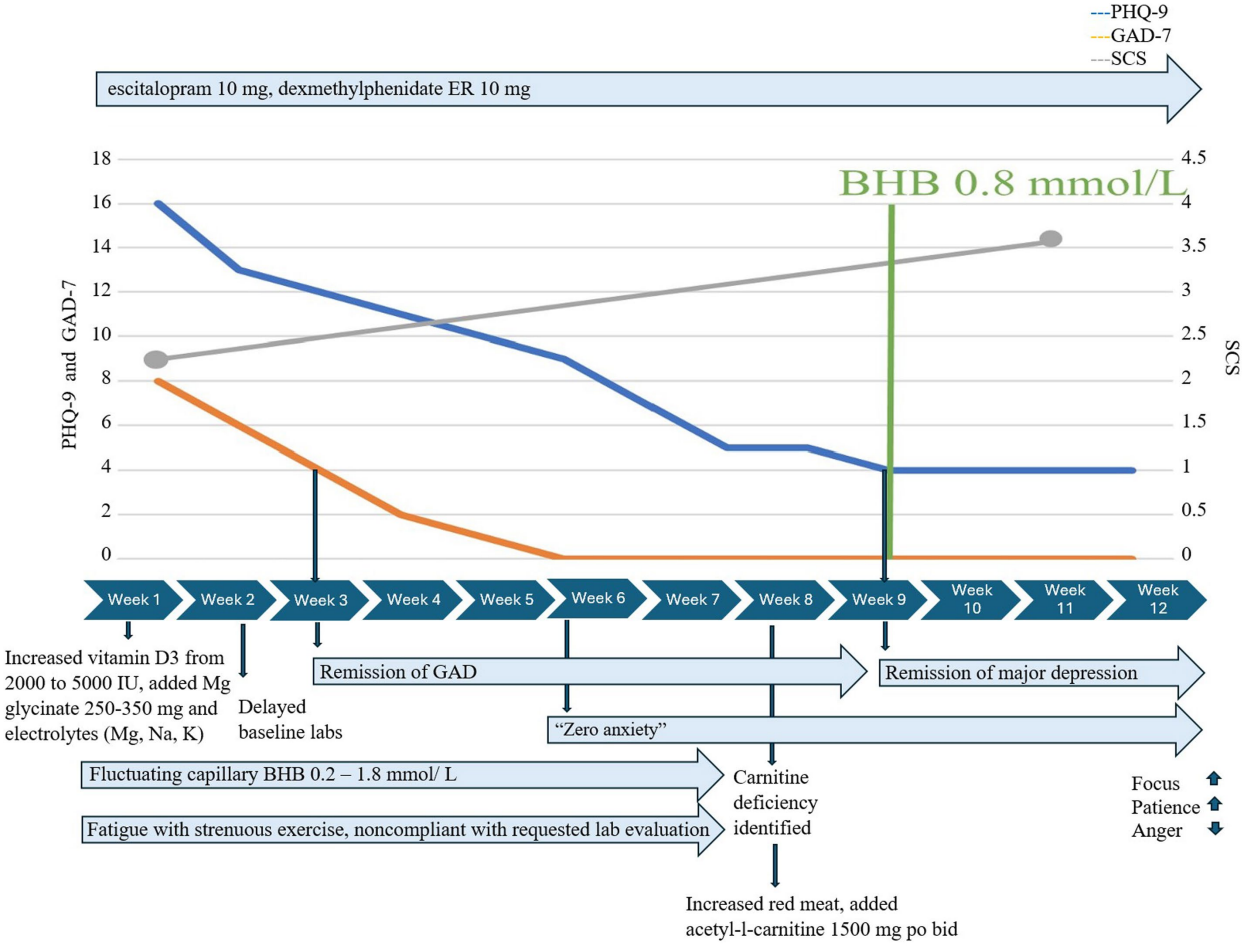
Case 2 KMT timeline.

Treatment of emergent fatigue with strenuous exercise required evaluation which identified low serum carnitine. He had delayed obtaining baseline serum carnitine at initiation and, despite symptoms, delayed recommended follow-up with follow-up serum acylcarnitine and urine carnitine when ordered. He obtained a baseline serum carnitine late in KMT at 8 weeks instead of before or during week 1. Carnitine esters were 21 mmol/L and the esterified/free ratio was 0.57. Additional carnitine evaluation was ordered: a complete acylcarnitine profile showed elevated acylcarnitine, C2 19.6 nmoL/mL, and mildly elevated OH-butyrlcarnitine, C4OH (0.08 nmoL/mL), and glutarylcarnitine, C5DC (0.07 nmoL/mL); further studies of urine carnitine showed elevated urine total carnitine (522 mmol/mg Cr) and urine free carnitine (211 nmol/mg Cr). In response, we advised increased red meat consumption and supplementation with acetyl-l-carnitine 1,500 mg po bid with meals; exercise fatigue resolved within days, and he quickly achieved consistent therapeutic ketosis with capillary BHB ≥ 0.8 mmol/L (Figure 2).

Despite inconsistent capillary BHB and GKI early in treatment, GAD-7 decreased from 8 to 4 within 2 weeks of KMT initiation and to 0 after an additional 4 weeks and remained at 0. Major depression was moderately severe at the initiation of KMT (PHQ-9 = 16), responded at 5.5 weeks (PHQ-9 = 8), and fully remitted at 9 weeks, coinciding with the first week in which he achieved consistent BHB ≥ 0.8 mmol/L/GKI 6.5 and had added acetyl-L-carnitine 1,500 mg po bid. Self-compassion increased from 2.7 to 4 over 12 weeks. He reported “increased mental focus,” more patience with coworkers and family, and stated he no longer felt “a general pull of anger all the time.”

Case 2 achieved optimal metabolic health in 12 weeks. He lost 21 lbs./9.5 kg, his BMI decreased from 27.8 to 24.9 kg/m^2^, % body fat decreased from 26.1% to 17.8%, his visceral fat level decreased from 10 to 6, and his HS-CRP normalized, decreasing from 2.5 to 1.

### Case 3

Case 3 was a 34-year-old single woman with a history of childhood adversity and trauma, PTSD, childhood-onset GAD, and recurrent severe major depressive disorder. She developed anorexia in adolescence and later binge eating disorder with weight fluctuations up to 100 lbs. and reported a long history of dietary attempts at weight loss, including the use of low-carb diets. She had been previously prescribed long trials of citalopram, risperidone, and ziprasidone, all of which were ineffective. ADHD, the inattentive subtype, was treated with a long-acting methylphenidate with intermittent compliance. Her medical history included irritable bowel syndrome, hypothyroidism, obstructive sleep apnea, chronic fatigue syndrome, and cholecystectomy. Medications included bupropion XL 150 mg po qam, duloxetine 60 mg po qd, lisdexamfetamine 20 mg po qam, NP thyroid 15 mg, and levothyroxine 25 mcg po qd. She was obese with a BMI 43.7 kg/m^2^, 52.2% body fat waist circumference > 35″, low HDL of 46, TG/HDL ratio of 2.1, elevated insulin resistance score of 67, elevated fasting insulin of 15 μIU/mL, and C-peptide of 2.08 ng/mL. HS-CRP was very high 6.4 mg/dL, with a dyslipidemic advanced lipid profile showing elevations in LDL particle number, small and medium LDL, and low and large HDL particles. BP was within normal limits.

Family history was notable for depression, generalized anxiety, and ADHD in first-degree relatives and unspecified psychosis in second-degree relatives; family medical history included obesity, type II diabetes mellitus, hyperlipidemia, and breast cancer in first-degree relatives.

**Figure 3:**
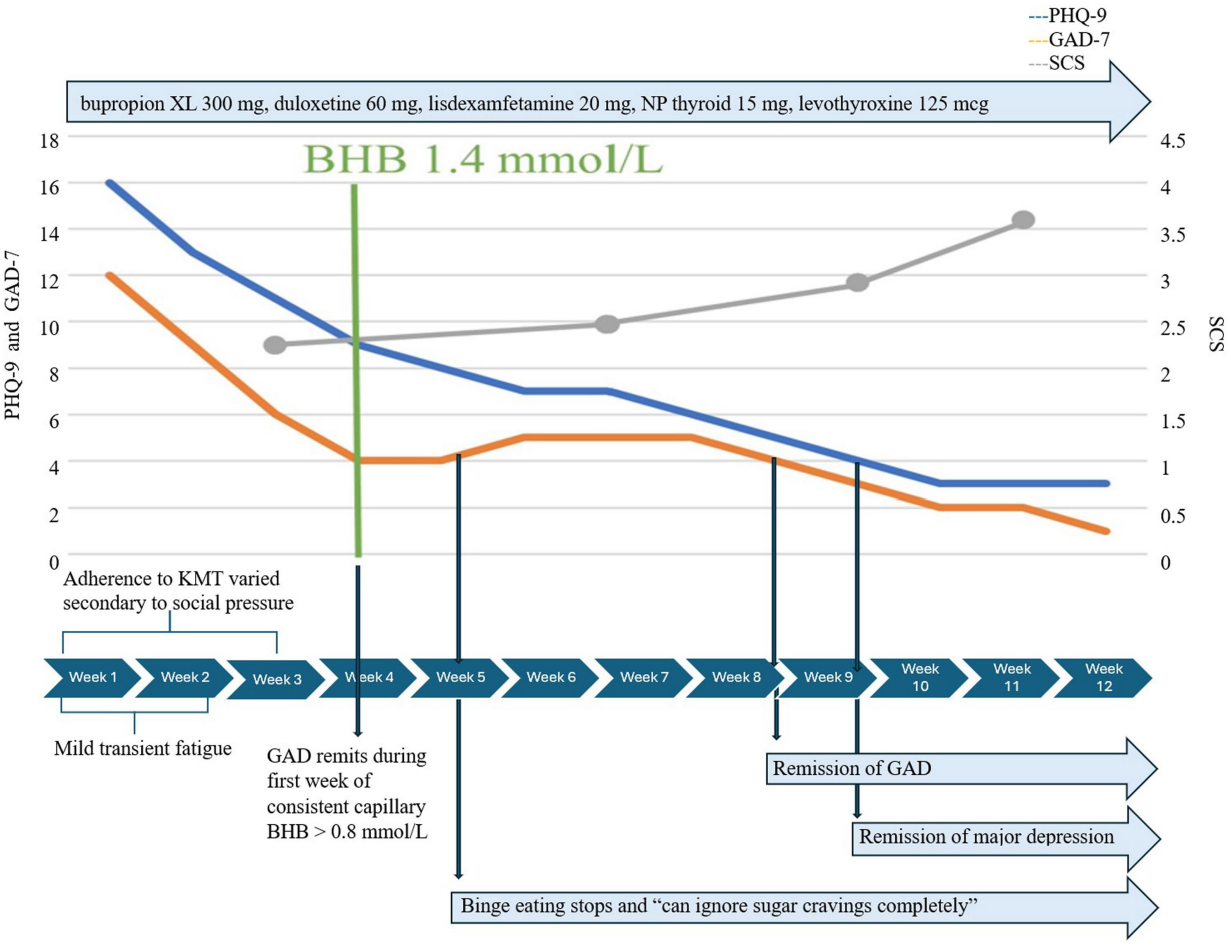
Case 3 KMT timeline.

She began KMT as adjunctive treatment to medication, which remained unchanged throughout 12 weeks. Initial mild transient fatigue resolved rapidly with no other adverse effects. Adherence to KMT was initially variable, affected by travel, holiday events, and family pressure to eat processed carbohydrates and desserts. She realized quickly that her lack of preparation for these events contributed to difficulties adhering to KMT and adopted simple strategies to prepare ahead. Initially, GAD-7 decreased from 12 to 6 within 2 weeks, even before consistent adherence to KMT. By week 3, when she achieved consistent therapeutic ketosis, the mean BHB was 1.4 mmol/L/GKI 4.2, and GAD-7 dropped to 4 for the first time (Figure 3). One week later, the mean BHB was 2.1 mmol/L/GKI 2.3, and she reported binge eating had stopped; she could “now ignore sugar cravings completely” and “no longer related to struggles with hunger and cravings.” Notably, KMT did not precipitate a return of anorexic thoughts, body preoccupation, or behaviors. She learned to navigate social situations and restaurants more easily while maintaining KMT.

Complete remission of depression occurred 5 weeks later, after a total of 8 weeks of consistent nutritional ketosis, and she said, “I do not have it anymore. I’ve just noticed I’m happy all the time, which is funny.” GAD remitted sooner, 5 weeks after consistent therapeutic ketosis. SCS increased from 3 to 4 over 12 weeks, and flourishing scale increased from 47 to 53.

Metabolically, despite insulin resistance and requiring 3 weeks to achieve consistent therapeutic ketosis, Case 3 lost 28.7 1bs/13.0 kg within 12 weeks, BMI decreased from 43.7 to 37.7 kg/m^2^, % body fat decreased from 52.2% to 48.9%, insulin resistance score decreased from 67 to 36, and hs-CRP decreased from 6.4 to 2.3.

None of these patients reported nausea, orthostasis, drowsiness, insomnia, agitation, or hypomania. There were no drug-KMT interactions identified, and none of the patients were treated with anticonvulsants, such as topiramate or zonisamide, which could have potentially increased the risk of nephrolithiasis.

## Discussion

Although KD was first shown to produce antidepressant effects and alleviate “behavioral despair” in preclinical studies more than 20 years ago (20), there is little clinical data regarding KD in major depression and anxiety disorders.

A retrospective analysis of 31 individuals with primary diagnoses of major depression (*N* = 7), bipolar II disorder (*N* = 13), and schizoaffective disorder (*N* = 12) who had failed to respond to conventional psychiatric care was treated with KD (75%–80% fat, 15%–20% protein, 5% carbohydrate) for 12 weeks in a psychiatric hospital (31). Of these patients, 22 were voluntarily admitted for the initiation of KD, and the remainder were offered KD during their inpatient hospital course. Change in depression was measured by HAM-D and MADRS in 6 of 7 patients with major depression and 12 of 13 patients with bipolar disorder. Notably, 100% of patients given the HAM-D showed statistically significant improvement in depressive symptoms (mean HAM-D decreased from 25.4 to 7.7; mean MADRS decreased from 29.6 to 10.1). However, serum ketones were not measured; urinary ketone measures were obtained once in 28 patients during the 12-week intervention; 18 patients (64%) showed positive urine ketones (31).

Bipolar depression was included in a recent randomized controlled pilot study assessing the safety and feasibility of KMT as adjunctive therapy, and reported safety and feasibility with excellent adherence and maintenance of ketosis (mean BHB 0.88 ± 0.99 mmol/L for 12 weeks) (32).

One case report utilized KD (65% fat, 25% protein, 10% carbs) with a time-restricted feeding window in a 65-year-old woman with major depression and type II diabetes and reported remission of depression (PHQ-9 17 to 0), normalization of HbA1c, decrease in estimated average glucose from 216 to 96 mg/dL, improvement in HOMA-IR from 9.4 to 2.3, and TG/HDL ratio from 4.7 to 1.2 over 12 weeks. The only measured serum BHB reported in the case was a mean of 1.5 mmol/L by week 12 (30).

A recent meta-analysis of low carbohydrate diets used in controlled trials that evaluated symptoms of depression and anxiety, not disorders, in varied metabolic and inflammatory conditions reported that the symptomatic response of these symptoms was inconclusive (33). The conclusions may not apply to KMTs; the meta-analysis was limited by grouping varied diets with higher carb intake and higher protein intake than usually associated with diets formulated to induce nutritional ketosis; serum or capillary BHB was not reported; and primary and secondary outcomes varied across studies.

In anxiety, preclinical research shows that exogenous ketone supplementation reduces anxiety behaviors (34). There is one case report of a self-administered Atkins Diet for weight loss in a woman with panic disorder (35) but no reports of KMT in panic disorder, OCD, or PTSD. Case reports of KD addressing anxiety describe two cases of decreased anxiety symptoms in a woman with women, one with bipolar I disorder and another with unspecified mood disorder, comorbid emotional dysregulation, body dysmorphic disorder, and eating disorder (36, 37), and one case report that describes the elimination of anxiety symptoms in a man with bipolar disorder (38). Anxiety and obsessive preoccupations improved in weight-restored anorexic women, in one case report describing complete remission of anorexia (39) and in a retrospective case series where animal-based KD was adopted without dietary prescription and monitoring (2); however, ketone measures were not reported. One small pilot trial where KD was followed by ketamine infusions reported a significant lessening of obsessive preoccupations in weight-recovered women with chronic anorexia; here, ketosis was measured by breath acetone (40).

Finally, all three patients had comorbid ADHD, which may be important. Approximately 65–89% of adults with ADHD experience one or more comorbid psychiatric conditions, and ADHD often occurs comorbid with anxiety and depression (41). Preclinical studies in murine models (42) of ADHD with hyperactivity suggest that KD may improve symptoms via alteration of the gut microbiome. Preclinical studies in dogs with epilepsy displaying ADHD-like behaviors treated with a medium-chain triglyceride KD have shown decreased pathological behaviors (43). Further research exploring the effect of KMT in humans with ADHD should be considered to understand the mechanisms of action and assess short- and long-term risks and benefits.

There is little research regarding the selection, implementation, and treatment course for KMT use as adjunctive or sole treatment in individual psychiatric conditions. Given the potential benefits of therapeutic nutritional ketosis and the restoration of metabolic health (44), there is a pressing need to identify the biological underpinnings of KD in psychiatric disorders and delineate factors associated with the successful adoption and adherence of KMT and responses in common psychiatric disorders such as depression and anxiety. In clinical practice and real-world settings, where patients often present with multiple comorbidities, consideration of KD can seem daunting to clinicians.

Despite that, this case series illustrates complete remission of both major depression and GAD in three adults with complex psychiatric comorbidity and previously unrecognized metabolic dysfunction using whole-food, animal-based personalized KMT. Anxiety responded first and time to remission occurred rapidly within 7–12 weeks, despite varied challenges, including preferences for time-restricted eating, slow adoption, inconsistent monitoring, and emergent fatigue during strenuous exercise, which occurred many weeks into KMT due to low serum carnitine and spontaneous reduction of protein intake rather than keto-adaptation or “keto-flu.” All patients improved metabolically, and one patient achieved optimal metabolic health (6).

These patients were representative of many adults in clinical psychiatric practice who present with persistent, serious symptoms interfering with several life domains. They each had five DSM-V psychiatric disorders: severe unipolar major depression, GAD, at least one other anxiety disorder (OCD, PTSD, and/or panic disorder), and ADHD, and two had binge eating disorder. They had all failed at least two previous adequate trials of medications and psychotherapy and were seeking relief. All had family histories of mood and anxiety disorders and documented metabolic disease in first-degree relatives. Extensive laboratory testing and bioimpedance evaluations were eye-opening because, although they were overweight, they were not aware of the extent to which they were already metabolically ill; we suspect it enhanced their motivation to adopt and maintain KMT.

This case series is limited by describing only three patients, which limits the generalizability of our results as well as the inherent selection bias, as they were interested in KMT after failing standard therapies. In addition, they were selected because their complex psychiatric comorbidity reflects the complexity seen in the majority of our outpatient psychiatric practice. This degree of complexity may limit the generalization of these findings, although it is important to note that outpatient clinical psychiatric practice as a whole has seen an increase in complex psychiatric comorbidity over the past two decades (44).

As a retrospective case series, there may be additional limiting and confounding factors, including the lack of a control group. Some rating scales and digital data are missing, which may impact the completeness of the analysis. Time to consistent nutritional ketosis and delays in obtaining necessary labs requiring intervention may have contributed to a longer time to response and remission; response and remission may occur earlier than reported here, and this deserves further research. Finally, it is not clear to what extent immersive treatment (see Supplementary material 1) and additional interventions during KMT, such as close digital monitoring, frequent clinical contact, group supports, and nature walks, contributed to the rapidity of response/remission of anxiety and depression, or to overall treatment success, independent of the biological effects of KMT, and which of these elements were most critical; more research is needed. Carefully designed prospective studies and randomized controlled trials providing higher levels of evidence are needed to examine the use of KMT and time to response and remission in individuals without comorbidity and determine the extent to which comorbidity may confound or alter these (8).

As metabolic psychiatry moves forward, we need both preclinical and larger, well-controlled clinical studies examining the pleiomorphic effects of ketone bodies in the brain and in the body to understand how we can best leverage whole foods to optimize brain energy, enhance genetic expression, reduce neuroinflammation, optimize metabolic health, and safeguard the promise of our future.

## Patient perspectives

“I’m very pleased with my progress in weight loss and focus and there is a significant improvement in energy, which are all at the best levels that they have ever been. My energy levels are as good as they were when I was an athlete in high school.” “I’ve been given more to handle [at work]” [Case 1].

“Sleeping well, feeling well overall, I feel a little less irritable. Something bad happened … and I handled it much more calmly than I ever would have.” “My ketone levels have been more consistent and I have not had any episodes of feeling weak during exercise since I started taking carnitine [with meals]” [Case 2].

“I’m not depressed anymore.” “I understand why things had gone wrong in the past with trying low carb diets on my own.” “The accountability and the community support is really important for me” [Case 3].

## Data availability statement

The datasets presented in this article are not readily available because of reasons of sensitivity, but de-identified data are available from the corresponding author upon reasonable request. Requests to access the datasets should be directed to LC, drcalabrese@loricalabresemd.com.

## Ethics statement

Ethical approval was not required for the studies involving humans because this is a retrospective review of three cases from the usual course of clinical practice. All individuals provided written informed consent for the treatment intervention described, and for publication of their data. All data has been de-identified to protect confidentiality. The studies were conducted in accordance with the local legislation and institutional requirements. The participants provided their written informed consent to participate in this study. Written informed consent was obtained from the individual(s) for the publication of any potentially identifiable images or data included in this article. Written informed consent was obtained from the participant/patient(s) for the publication of this case report.

## Author contributions

LC: Conceptualization, Data curation, Investigation, Methodology, Supervision, Writing – original draft, Writing – review & editing. RF: Conceptualization, Data curation, Investigation, Writing – original draft, Writing – review & editing. MG: Investigation, Writing – review & editing.
