# Supplementary material for: Complete remission of depression and anxiety using a ketogenic diet: case series
Source: Front Nutr. 2024 May 14;11:1396685. doi: 10.3389/fnut.2024.1396685 (PMC11182043; doi:10.3389/fnut.2024.1396685)
Supplement: Supplementary file 1 [file Data_Sheet_1.pdf]

THERAPEUTIC INTERVENTION

Clinical Setting

Ketogenic metabolic therapy was provided through a full-service outpatient psychiatric practice offering psychopharmacology, interventional psychiatry, psychotherapy, and a specialized metabolic psychiatry program, staffed by a psychiatrist experienced in metabolic therapies, a registered ketogenic dietitian-nutritionist, and a LPN/medical assistant. All healthcare assessment, education, intervention, community and lifestyle supports, and medical treatment was provided by licensed clinical staff under one roof, in-person and virtually. A Metabolic Psychiatry Registry was established at the initiation of the metabolic psychiatry program to collect demographic, clinical, treatment outcome, and safety data during the regular conduct of clinical care.

Comprehensive Metabolic Psychiatry Evaluation

Three adult outpatients, ages 32-36, with unipolar major depression, generalized anxiety, and complex psychiatric comorbidity, refractory to standard of care therapy, were selected for review from our Metabolic Psychiatry Registry. They reflect the complex psychiatric comorbidity often seen in real-world psychiatric practice. Each patient had five concurrent DSM-V psychiatric disorders, including moderately severe major depression (MDD), generalized anxiety disorder (GAD), and at least one other anxiety disorder (obsessive compulsive disorder, panic disorder, and/or post traumatic stress disorder). Two had comorbid binge eating disorder; all had comorbid attention deficit hyperactivity disorder, with active symptoms at the time of presentation. No patient had a history of hypomania, mania, psychosis, or substance use disorder.

Patients underwent a comprehensive metabolic psychiatry evaluation over the course of 3 visits, which included intensive psychiatric evaluation, medical evaluation, detailed nutritional assessment and lifestyle assessment, and collaborative joint feedback. Joint feedback was used to identify shared treatment goals and discuss treatment options, including the risks/benefits/alternatives to and appropriateness of ketogenic metabolic therapy, and to outline use of KMT in a comprehensive metabolic program to target anxiety and depressive symptoms.

Medical records and recent labs were reviewed to verify medical history and to rule out pancreatitis, liver disease, pyruvate carboxylase deficiency, fatty acid oxidation defects, primary carnitine deficiency, palmitoyl transferase I or II deficiency, carnitine translocase deficiency, and acute intermittent porphyria, as well as to identify medical conditions requiring attention before and during KMT.

Comprehensive screening labs were ordered, including comprehensive metabolic profile, HbA1c, uric acid, carnitine, calcium, magnesium, zinc, advanced lipid profile with inflammation including HS-CRP, insulin resistance panel with C-peptide, Vitamin B12, methylmalonic acid/homocysteine, Vitamin D 1,25(OH), CBC, iron and total iron binding capacity, ferritin, thyroid panel with TSH, T3 reverse. Bioelectrical impedance analysis (BIA) of lean body mass,

skeletal muscle mass, body fat mass, and visceral fat was obtained at baseline and throughout treatment at regular intervals.

All patients engaged in detailed discussion about the risks and benefits of personalized KMT, alternatives, and the importance of close psychiatric and metabolic monitoring via in-person and virtual visits, and using digital tools including daily capillary ketone/glucose testing with Keto-Mojo® GK+ Blood Glucose and β-Ketone Dual Monitoring System (ketone/glucose correlation coefficients to serum of 0.9927/0.9974) measuring d-beta-hydroxybutyrate (BHB), glucose, and glucose/ketone index (GKI) and meal photo journaling. All provided written informed consent for KMT and to allow publication of their de-identified data.

<u>Animal-Based Ketogenic Diet</u>

A personalized animal-based ketogenic metabolic therapy (KMT) was developed for each patient by a ketogenic registered dietitian using ratios of 1.5:1 to 2.5:1 (fat: protein + carbohydrates), with macronutrient ranges of approximately 75% fat, 20% protein, 5% or less carbohydrate. Each KMT incorporated individual food preferences and sensitivities, lifestyle concerns, taking into account shopping, meal planning and prep, and work/family schedules. Favorite recipes were adapted and new recipes and meal plans were provided.

Patients implemented KMT with a ratio of 1.5:1 at home, using whole foods to meet fat, protein and carbohydrate needs. Commercial ketogenic foods and food delivery services were not utilized. Animal-based proteins were employed due to high bioavailability and superior amino-acid profiles. Processed seed oils were intentionally avoided; added sugars, non-nutritive sweeteners and sweetening agents were eliminated. Exogenous ketone supplements were not utilized. Adequate hydration with liberal use of salt was stressed. Other nutritional strategies to potentially reduce anxiety, including caffeine reduction, use of prebiotics and probiotics, and supplementation with magnesium were not employed.

Multiple treatment elements, or touchpoints, were provided during KMT. These included biweekly individual visits in-person and virtually; a capillary blood ketone/glucose monitor (Keto-Mojo® GK+ Blood Glucose and β-Ketone Dual Monitoring System) and digital tools for daily capillary BHB/glucose/GKI measurements; digital tools for photo journaling; regular bio-impedance measures every 2-4 weeks; laboratory follow-ups as indicated; group meetings twice weekly; friends and family group meetings weekly; lifestyle interventions including nature walks and talks several times a week with the ketogenic registered dietitian and at regular intervals with psychiatrist; and community building. Screening for potential side effects of KMT was available daily through digital apps, and occurred twice weekly during clinical visits. Psychiatric follow-up visits were scheduled as needed. Successful adherence to KMT was documented by meal photo journaling and defined as consistent daily maintenance of mean capillary BHB $\geq$ 0.8 mmol/ L and GKI $<$ 6. Although BHB $\geq$ 0.5 mmol/L is often used to define ketosis, we elected to use a mean BHB $\geq$ 0.8 mmol/L to define consistent nutritional ketosis in our patients in order to maintain capillary BHB $>$ 0.5 mmol/L.
